# Supplementary material for: Content-rich biological network constructed by mining PubMed abstracts
Source: BMC Bioinformatics. 2004 Oct 8;5:147. doi: 10.1186/1471-2105-5-147 (PMC528731; doi:10.1186/1471-2105-5-147)
Supplement: Additional File 2 — The original results of the above study (non-essential files are deleted to keep the file size under the limit set by BMC bioinformatics). [file 1471-2105-5-147-S2.bz2 › chilibotAdditionalFile2/dip10/104ID9362454E434/html/BCL2L1_BAK1.html]

 


 **BCL2L1** and **BAK1** 
  
Found 265 abstracts in PubMed, retrieved 10.  
 

 What does Google say? 
 PDF only 
| .edu only 

---

**Interactive relationship** (e.g. stimulation, inhibition, etc)

**Stimulatory relationship**- Unlike Bcl 2, Bak  [ **BAK1** ]  stimulates several apoptosis pathways, and by interacting with Bcl 2 or Bcl XL  [ **BCL2L1** ] , it counteracts the anti cell death effect.  Ref: 12836337 Nucleic Acids Res Suppl, 2001
**Neutral relationship**- gossypol induced apoptosis triggers down regulation of anti apoptosis Bcl 2 members Bcl X  [ **BCL2L1** ]  L, Bag 1 and Mcl 1, up regulation of pro apoptosis Bcl 2 member Bak  [ **BAK1** ] , activation of caspase,, and 9, up regulation of Apaf 1, release of cytochrome c cyto c from mitochondria, and activation of both DFF45 and PARP.  Ref: 12818369 Biochem Pharmacol, 2003

**Non-interactive relationship** (e.g. studied together, co-existance, homology, etc.)

- HSpin1 bound to Bcl 2 and apoptosis regulator Bcl X  [ **BCL2L1** ]  Bcl x  [ **BCL2L1** ]  L, but not to proapoptotic members such as Bcl 2 associated X protein and Bcl 2 homologous antagonist killer  [ **BAK1** ] , in cells treated with TNF alpha.  Ref: 12815463 Cell Death Differ, 2003
- Furthermore, Bcl 2 family members Bad, Bak  [ **BAK1** ] , and Bcl xS  [ **BCL2L1** ]  protein levels were increased in FHIT transfected clones when compared with Panc 1 cells.  Ref: 12890991 Pancreatology, 2003
- We also analyzed the relationship between TCF 4 gene splicing isoforms, proliferation proliferating cell nuclear antigen labeling index, and apoptosis antiapoptotic factors Bcl 2 and Bcl x  [ **BCL2L1** ]  L, proapoptotic factors Bak  [ **BAK1** ]  and Bax, and caspase 3 in RCC samples.  Ref: 12796377 Clin Cancer Res< MedlineTA>Clin Cancer Res, 2003
- PURPOSEWe hypothesized that apoptosis is a downstream event in erectile dysfunction, and pro apoptotic Bak  [ **BAK1** ]  and Bax and anti apoptotic Bcl 2 and Bcl x  [ **BCL2L1** ]  factors are involved in the etiology of diabetes induced erectile dysfunction.  Ref: 12796708 J Urol< MedlineTA>J Urol, 2003
